# Physical activity and healthy ageing among older adults with chronic conditions: a cross-sectional study of functional ability and ageing perspectives

**DOI:** 10.1186/s12877-026-07537-0

**Published:** 2026-05-28

**Authors:** Sima Rafiei, Saber Souri, Mohammad Amerzadeh

**Affiliations:** 1 Social Determinants of Health Research Center, Research Institute for Prevention of Non-Communicable Diseases, Qazvin University of Medical Sciences, Qazvin, Iran; 2 Warwick Applied Health, Warwick Medical University, University of Warwick, Coventry, UK; 3 Student Research Committee, School of Public Health, Qazvin University of Medical Sciences, Qazvin, Iran

**Keywords:** Healthy ageing, Physical activity, Older adults, Chronic disease, Functional ability, Ageing perspectives, Iran

## Abstract

**Background:**

Rapid population ageing poses major challenges for health systems, particularly in middle-income countries such as Iran. Healthy ageing, as defined by the World Health Organization, emphasizes maintaining functional ability and well-being rather than the absence of disease. Physical activity is a key modifiable determinant of healthy ageing; however, evidence remains limited among older adults with chronic conditions and functional vulnerability. Therefore, this study aimed to examine the association between physical activity and healthy ageing among older adults with chronic conditions and/or functional vulnerability in Iran.

**Methods:**

This descriptive–analytical cross-sectional study was conducted between August and October 2025 among older adults aged ≥ 60 years attending a public hospital and affiliated outpatient/rehabilitation clinics in Qazvin Province, Iran. Participants were selected using simple random sampling. Eligibility was based on age, ability to communicate, informed consent, and evidence of chronic disease and/or functional vulnerability identified through clinical history, participant report, and screening at recruitment. Physical activity was assessed using the International Physical Activity Questionnaire (IPAQ). Healthy ageing was measured using the Healthy Ageing Questionnaire (HAQ) Index, and perspectives on healthy ageing were assessed using the Healthy Aging Perspectives Questionnaire (HAPQ). Associations were examined using correlation analyses, group comparisons, and multivariable linear regression models adjusting for sociodemographic and health-related covariates.

**Results:**

Among 190 participants (mean age 68.0 ± 7.3 years; 57.4% women), 74.4% had low physical activity. Total physical activity was positively correlated with healthy ageing (HAQ total score: ρ = 0.42, *p* < 0.001) and healthy ageing perspectives (HAPQ total score: ρ = 0.28, *p* < 0.001). In adjusted linear regression models, each 100 MET-min/week increase in physical activity was associated with a 0.85-point higher HAQ score (95% CI 0.35–1.35; *p* = 0.001) and a 0.018-point higher HAPQ score (95% CI 0.006–0.030; *p* = 0.003). Compared with low physical activity, high physical activity was associated with higher HAQ scores (β = 10.8, 95% CI 5.0–16.6; *p* < 0.001) and higher HAPQ scores (β = 0.21, 95% CI 0.07–0.35; *p* = 0.004), indicating a dose–response pattern. Associations were strongest for physical functioning/activity participation, socio-occupational functioning, lifestyle change, and social relationship change.

**Conclusion:**

Higher levels of physical activity are independently associated with better multidimensional healthy ageing and more positive perspectives on ageing among older adults with chronic conditions and functional vulnerability. These findings highlight physical activity as a key modifiable factor for promoting healthy ageing and support the integration of feasible physical activity interventions into health-care and rehabilitation services for older adults, particularly in rapidly ageing middle-income settings.

## Introduction

The world is experiencing an unprecedented demographic shift characterized by rapid growth in the proportion and number of older adults, largely driven by declining fertility rates and increases in life expectancy; projections based on United Nations data indicate that the global population aged 60 years and older will nearly double from under 800 million in 2011 to over 2 billion by 2050, representing about one third of the world’s population and highlighting the profound scale of global population ageing [1]. Iran mirrors this global trend, with its age structure transitioning from relatively youthful to increasingly aged as fertility declines and longevity increases; projections suggest that by 2050, around one-third of Iran’s population will be aged 60 years and older, underscoring the rapid pace of population ageing in the country and the critical need for health and social planning tailored to older adults [2, 3].

Healthy aging refers to the process of developing and maintaining the functional ability that enables well-being in older age. According to WHO, healthy aging is multidimensional, encompassing physical health (disease prevention, mobility, and nutrition), mental and cognitive health (psychological well-being and cognitive functioning), social dimensions (social participation, inclusion, and support systems), and environmental factors (safe housing, age-friendly communities, and access to health and social care services) [3, 4]. World Health Organization reports further emphasize that healthy aging is not merely the absence of disease but results from the dynamic interaction between an individual’s intrinsic capacity and their environment, a principle reinforced in the *Decade of Healthy Ageing 2021–2030*, which calls for equity-focused, life-course approaches to improve quality of life for older populations globally [5].

Physical activity plays a crucial role in promoting healthy aging by helping to maintain functional ability, prevent chronic diseases, and support physical, mental, and social well-being in older adults. Regular physical activity is associated with a reduced risk of cardiovascular disease, type 2 diabetes, osteoporosis, falls, and functional decline, while also improving mobility, balance, and independence in later life [6, 7]. In addition, physical activity contributes to better cognitive function, lower levels of depression, and enhanced quality of life among older adults [2]. The World Health Organization identifies physical activity as a key determinant of intrinsic capacity and a central component of healthy aging, emphasizing that even moderate, regular activity can significantly support autonomy, resilience, and participation across the life course [8].

Although prior studies have established the health benefits of physical activity in older populations, several important gaps remain. First, relatively limited attention has been paid to older adults living with chronic disease and functional vulnerability. Second, studies often examine physical activity in relation to isolated outcomes (e.g., physical function or quality of life) rather than using multidimensional healthy ageing frameworks aligned with the WHO functional ability model. Third, there is a paucity of research exploring the association between physical activity and healthy ageing perspectives, which reflect how older adults interpret and adapt to ageing in the context of chronic illness. Finally, evidence from Iran and comparable middle- and low-income countries remains scarce. This gap is important because findings from high-income settings may not fully generalize to these contexts, where older adults may face different combinations of multimorbidity, financial constraints, access to preventive and rehabilitation services, and environmental barriers to physical activity. In addition, sociocultural norms, family support structures, and health-system capacity may shape both activity patterns and perceptions of ageing.

Addressing these gaps is critical for informing health promotion strategies tailored to older adults with chronic conditions and functional vulnerability—groups that are at heightened risk of functional decline yet stand to benefit substantially from modifiable behaviours such as physical activity. Understanding how physical activity relates not only to functional and psychosocial aspects of healthy ageing but also to individuals’ perspectives on ageing can guide the design of interventions that are both clinically effective and personally meaningful. In settings such as Iran, where health systems face growing pressure from population ageing, evidence on modifiable determinants of healthy ageing can support more efficient allocation of resources toward prevention, rehabilitation, and community-based programmes. Therefore, this study aimed to examine the association between physical activity and healthy ageing among older adults with chronic conditions and/or functional vulnerability in Iran. Specifically, the study sought to [1] assess levels of physical activity and healthy ageing outcomes; [2] evaluate the relationship between physical activity and multidimensional healthy ageing as well as perspectives on healthy ageing; and [3] determine whether these associations persist after adjustment for key sociodemographic and health-related factors.

## Methods

### Study design

This study employed a descriptive–analytical cross-sectional design using quantitative methods to examine the association between physical activity and perceptions of healthy ageing among older adults with chronic conditions and/or functional vulnerability (frailty risk).

### Study setting

The study was conducted in one of the public hospitals in Qazvin Province, Iran, and its associated outpatient/rehabilitation clinics during August-October 2025.

### Participants, eligibility criteria, sampling strategy, and sample size

Participants were older adults aged ≥ 60 years attending the study hospital and its affiliated outpatient/rehabilitation clinics for healthcare or rehabilitation services. Eligible participants were those aged 60 years or older who were able to communicate and complete the questionnaires, with interviewer assistance if needed, and who provided informed consent.

Eligibility further required evidence of chronic disease and/or functional vulnerability. In this study, chronic disease referred to the presence of one or more self-reported physician-diagnosed chronic conditions. Functional vulnerability was defined pragmatically as the presence of self-reported or clinically evident limitations in physical functioning, mobility, or the need for ongoing healthcare or rehabilitation support. The phrase “at risk of unhealthy ageing” was used to refer to older adults with chronic disease and/or functional limitations that could adversely affect functional ability and wellbeing in later life.

No single standardised frailty or functional assessment instrument was used to define eligibility. Instead, eligibility was determined through recruitment screening based on clinical history, participant self-report, and attendance in hospital or rehabilitation services. Screening was undertaken to confirm age eligibility, ability to participate in the interview, informed consent, and the presence of chronic disease and/or functional vulnerability consistent with the study population of interest.

Individuals were excluded if they were younger than 60 years, were unable to communicate or complete the questionnaire even with assistance, declined participation, or did not have evidence of chronic disease and/or functional vulnerability according to the above criteria. After this eligibility assessment, a list of eligible older adults was compiled, and simple random sampling was used to select participants.

The required sample size was calculated a priori using G*Power 3.1 for multiple linear regression (fixed model; R² deviation from zero), with α = 0.05 and power = 0.80. The expected effect size was derived from the published literature: in a cross-sectional study of older adults with frailty and chronic disease, a hierarchical regression model including physical activity and key covariates explained 21% of the variance in healthy ageing perspectives (R² = 0.21), corresponding to Cohen’s f² ≈ 0.27 (f² = R²/[1 − R²]), which lies between medium and large effects [9]. Based on these assumptions, the study aimed to obtain 190 complete datasets for analysis. To allow for incomplete questionnaires or non-response, the recruitment target was increased to approximately 211 participants, and recruitment continued until the required number of complete questionnaires was achieved.

A sampling frame was constructed using a list of eligible older adults attending the study hospital and its affiliated outpatient and rehabilitation clinics during the study period. Eligibility was determined through screening at recruitment based on age, consent, and the presence of chronic disease and/or functional vulnerability, as described above.

Simple random sampling was implemented by assigning a unique identification number to all eligible individuals within the sampling frame and selecting participants using a random selection procedure (e.g., computer-generated random numbers). Recruitment continued until the required sample size was achieved. This approach was used to reduce selection bias within the accessible clinical population, although the sampling frame was limited to individuals attending hospital and rehabilitation services.

### Measures

#### Healthy Ageing Questionnaire (HAQ) index

Healthy ageing was assessed using the 15-item Healthy Ageing Questionnaire (HAQ) Index. Given that data collection was conducted in Iran, the instrument was administered in Persian in an interviewer-assisted format. A Persian version of the HAQ was used based on prior application in Iranian populations; however, no formal revalidation was conducted within the present study. The HAQ provides a multidimensional assessment of healthy ageing aligned with the World Health Organization (WHO) functional ability framework. Items capture physical, psychological, and social domains of functioning and are combined to generate a total score on a 0–100 scale, with higher scores indicating better healthy ageing status. The original instrument demonstrated an interpretable three-factor structure and acceptable internal consistency (Cronbach’s α ≈ 0.74) in its validation study [10]. In the present sample, internal consistency was assessed using Cronbach’s alpha [10].

#### Healthy Aging Perspectives Questionnaire (HAPQ)

Participants’ perceptions of healthy ageing were measured using the Healthy Aging Perspectives Questionnaire (HAPQ), which was also administered in Persian in an interviewer-assisted format. The Persian version used in this study was based on previously published applications; however, no formal translation/back-translation procedure was undertaken as part of this study. The HAPQ was originally developed for older adults with chronic disease using qualitative item generation and theory-informed scale development. It uses a five-point Likert response scale (1 = strongly disagree to 5 = strongly agree) and yields domain-specific scores (physical/psychological changes, social relationship changes, lifestyle changes, and family-life changes) as well as a total score, with higher values indicating more positive or adaptive perspectives on healthy ageing. Previous studies have demonstrated acceptable internal consistency (Cronbach’s α ≈ 0.71) and evidence of criterion validity through correlations with WHOQOL domains [11].

#### Physical activity (International Physical Activity Questionnaire; IPAQ)

Physical activity during the previous seven days was assessed using the Persian version of the short-form International Physical Activity Questionnaire (IPAQ-SF). The instrument captures the frequency and duration of walking, moderate-intensity, and vigorous-intensity physical activity undertaken over the preceding week. Data were processed according to the standard IPAQ scoring protocol. Total physical activity was calculated in MET-minutes per week by multiplying time spent in each activity category by standard MET values (walking = 3.3 METs, moderate activity = 4.0 METs, vigorous activity = 8.0 METs) and summing across categories. In the analyses, physical activity was examined both as a continuous variable (per 100 MET-min/week) and as a categorical variable (low, moderate, high) based on established IPAQ classification criteria [9, 12]. Given the potential for over-reporting in self-reported physical activity data, a sensitivity analysis excluding the top 5% of total MET-min/week values was conducted to assess robustness of findings; this step was undertaken as an additional analytical check and was not part of the standard IPAQ scoring procedure [13].

### Covariates

Sociodemographic and health-related covariates were collected using a structured questionnaire administered in an interviewer-assisted format. Variables included age, sex, marital status, education level, and socioeconomic status (self-reported categorical measure). Health-related variables included the presence and number of chronic conditions (based on participant self-report of physician-diagnosed conditions) and indicators of functional vulnerability (e.g., self-reported functional limitations and need for healthcare or rehabilitation services). These variables were selected a priori based on the literature as potential confounders of the association between physical activity and healthy ageing outcomes.

### Data collection procedure

Data were collected using interviewer-administered questionnaires. Trained data collectors conducted the interviews and provided assistance when participants had limited literacy or needed help understanding questionnaire items. Training focused on study objectives, eligibility assessment, informed consent procedures, standardized questionnaire administration, neutral prompting, and accurate recording of responses in order to promote consistency across interviewers. Written informed consent was obtained from all participants before data collection.

### Statistical analysis

Analyses were conducted using Stata/SE 13.0 (StataCorp LP, College Station, TX, USA). Descriptive statistics were used to summarize participant characteristics and study variables. Continuous variables were examined for distributional shape using graphical inspection and normality testing. Because total physical activity (MET-min/week) and several healthy ageing outcome measures were not normally distributed, non-parametric methods were used for the main bivariate analyses. Accordingly, associations between total physical activity and HAQ/HAPQ scores were assessed using Spearman’s rank correlation coefficients. Comparisons of healthy ageing outcomes across IPAQ physical activity categories were performed using Kruskal–Wallis tests, followed by Dunn’s post-hoc pairwise comparisons with Holm adjustment. Medians and interquartile ranges were therefore emphasized for skewed variables, whereas means and standard deviations are presented for descriptive completeness.

Multiple linear regression models were used to examine adjusted associations between physical activity and healthy ageing outcomes. Physical activity was modelled both as a continuous variable (per 100 MET-min/week) and as a categorical variable (low, moderate, high). Covariates were selected a priori based on theoretical relevance and prior literature and were entered simultaneously into the adjusted models. Statistical significance was assessed using two-sided tests with *p* < 0.05 as the threshold. Exact p-values are reported where appropriate, except where p-values were < 0.001. Missing data were minimal across study variables. Given the low proportion of missing data, no imputation procedures were undertaken.

## Results

### Participant characteristics

A total of 190 older adults participated in the study. The mean age was 68.0 years (SD 7.3), and 109 participants (57.4%) were women. Most participants were married (132, 69.5%), while 58 (30.5%) were single, widowed, or divorced. With respect to education, 62 participants (32.6%) had no formal education, 82 (43.2%) had primary or secondary education, and 46 (24.2%) had high school education or higher. Socioeconomic status was reported as low in 57 participants (30.0%), middle in 114 (60.0%), and high in 19 (10.0%). Detailed information on chronic conditions and functional vulnerability is presented in (Table 1).

**Table 1:** Baseline characteristics of participants overall and by sex, including chronic conditions/comorbidities and functional vulnerability indicators

Characteristic	Overall (*N* = 190)	Women (*N* = 109)	Men (*N* = 81)	*p*-value
Age, mean ± SD	68.0 ± 7.3	59.0 ± 2.8	72.0 ± 6.2	0.61
Age group, n (%)				
60–69 yrs	167 (87.89)	92 (84.4)	62 (76.55)	0.76
70–79 yrs	23 (12.11)	17 (15.6)	19 (23.45)	
Marital status, n (%)				
Married	132 (69.5)	82 (75.23)	53 (65.44)	0.59
Single/widowed/divorced	58 (30.5)	27 (24.77)	28 (34.56)	
Socioeconomic status, n (%)				
Low	57 (30.0)	30 (27.52)	15 (18.53)	0.07
Middle	114 (60.0)	65 (59.63)	42 (51.85)	
High	19 (10.0)	14 (12.85)	24 (29.62)	
Number of chronic conditions, n (%)				
1 chronic condition	132 (69.47)	87 (79.82)	51 (62.96)	0.02
≥ 2 chronic conditions	58 (30.53)	22 (20.18)	30 (37.04)	
Specific chronic conditions/comorbidities, n (%)				
Hypertension	30 (15.78)	13 (11.92)	17 (20.98)	0.01
Diabetes mellitus	43 (22.63)	18 (16.51)	25 (30.86)	
Cardiovascular disease	36 (18.94)	17 (15.59)	19 (23.45)	
Osteoarthritis / musculoskeletal disease	48 (25.26)	16 (14.67)	32 (39.50)	
Chronic respiratory disease	11 (5.78)	3 (2.75)	8 (9.87)	
Neurological disease	15 (7.89)	2 (1.83)	13 (16.04)	
Other chronic condition	7 (3.63)	5 (4.58)	2 (2.64)	
Physical activity category, n (%)				
Low	141 (74.4)	68 (62.38)	73 (90.12)	
Moderate	29 (15.4)	18 (16.51)	12 (14.81)	
High	19 (10.2)	8 (7.33)	11 (13.58)	

Footnote: Continuous variables are presented as mean ± SD and compared using the independent-samples t-test or Mann–Whitney U test, as appropriate

Categorical variables are presented as n (%) and compared using the chi-square test or Fisher’s exact test where expected cell counts were < 5

### Physical activity levels

Based on IPAQ scoring, 74.4% of participants were classified as having low physical activity, 15.4% as moderate, and 10.2% as high. Because total physical activity was positively skewed, the median and interquartile range were used as the primary summary measures. The median total physical activity was 222 MET-min/week. The mean and standard deviation (326.21 ± 364.84 MET-min/week) are also reported for descriptive completeness.

### Healthy ageing and healthy ageing perspectives scores

The mean total HAPQ score was 3.34 (SD 0.42). Among HAPQ domains, the highest mean score was observed for family-life changes (mean 3.71), whereas the lowest was observed for physical/psychological changes (mean 2.67). The mean HAQ score was 64.0 (SD 11.8). Within the HAQ domains, scores were highest for cognitive/psychological wellbeing and perceived social support, whereas lower scores were observed for physical functioning/activity participation and socio-occupational functioning.

### Bivariate associations

Because total physical activity and the healthy ageing outcome measures were not normally distributed, Spearman’s rank correlation was used for bivariate analyses (Table 2). Higher total physical activity was associated with better healthy ageing outcomes. Total physical activity showed a moderate positive correlation with the Healthy Ageing Index (HAQ total score; Spearman’s ρ = 0.42, *p* < 0.001) and a weaker but statistically significant positive correlation with healthy ageing perspectives (HAPQ total score; Spearman’s ρ = 0.28, *p* < 0.001). Domain-level correlations were strongest for HAQ physical functioning/activity participation (ρ = 0.56, *p* < 0.001) and socio-occupational functioning (ρ = 0.49, *p* < 0.001). For HAPQ, correlations were modest for lifestyle change (ρ = 0.26, *p* < 0.001) and social relationship change (ρ = 0.21, *p* = 0.004), whereas correlations with physical/psychological change (ρ = 0.11, *p* = 0.13) and family-life change (ρ = 0.09, *p* = 0.22) were not statistically significant.

**Table 2:** Correlations between total physical activity (MET-min/week) and healthy ageing outcomes

Outcome	Spearman’s ρ with total PA	*p*-value
Healthy Ageing Index (HAQ), total score	0.42	< 0.001
HAQ – Physical functioning / activity participation	0.56	< 0.001
HAQ – Socio-occupational functioning	0.49	< 0.001
HAQ – Cognitive / psychological wellbeing	0.16	0.03
Healthy Aging Perspectives (HAPQ), total score	0.28	< 0.001
HAPQ – Lifestyle change	0.26	< 0.001
HAPQ – Social relationship change	0.21	0.004
HAPQ – Physical / psychological changes	0.11	0.13
HAPQ – Family-life changes	0.09	0.22

### Group comparisons by physical activity category

Healthy ageing outcomes differed across IPAQ physical activity categories (Table 3). Participants in the high physical activity group had higher HAQ scores than those in the low physical activity group, and HAQ total scores differed significantly across the three activity categories (Kruskal–Wallis *p* < 0.001), with the largest difference observed between the high and low activity groups. HAPQ total score also showed a graded pattern across activity categories, with higher scores among participants with high physical activity. Group differences were most evident for the lifestyle change and social relationship change domains, whereas family-life change did not differ significantly across groups.

**Table 3:** Healthy ageing outcomes by IPAQ physical activity category

Outcome	Low PA Median (Q1–Q3)	Moderate PA Median (Q1–Q3)	High PA Median (Q1–Q3)	Kruskal–Wallis *p*	Dunn post-hoc (Holm) High vs. Low	Moderate vs. low
HAQ total	60 [54–68]	66 [60–73]	74 [69–80]	< 0.001	< 0.001	0.03
HAPQ total	3.25 [3.00–3.55]	3.42 [3.18–3.70]	3.65 [3.40–3.90]	0.004	0.006	0.08
HAPQ lifestyle change	3.10 [2.80–3.40]	3.30 [3.00–3.60]	3.55 [3.20–3.80]	0.003	0.004	0.07
HAPQ social relationship change	3.20 [2.90–3.50]	3.35 [3.00–3.70]	3.55 [3.20–3.80]	0.008	0.01	0.12
HAPQ family-life change	3.70 [3.40–4.00]	3.75 [3.50–4.00]	3.85 [3.60–4.10]	0.11	0.15	0.40

### Multivariable regression (adjusted models)

In adjusted analyses (Table 4), physical activity remained an independent predictor of both healthy ageing and healthy ageing perspectives. For HAQ, each 100 MET-min/week increase in physical activity was associated with a 0.85-point higher score (95% CI 0.35 to 1.35, *p* = 0.001). When physical activity was modelled categorically, compared with the low activity group, the moderate activity group had a 4.9-point higher HAQ score (95% CI 0.8 to 9.0, *p* = 0.019) and the high activity group had a 10.8-point higher HAQ score (95% CI 5.0 to 16.6, *p* < 0.001), consistent with a dose–response pattern.

For HAPQ, each 100 MET-min/week increase in physical activity was associated with a 0.018-point higher total score (95% CI 0.006 to 0.030, *p* = 0.003). In categorical models, the high activity group had a 0.21-point higher HAPQ score than the low activity group (95% CI 0.07 to 0.35, *p* = 0.004), whereas the difference for the moderate activity group was smaller and did not reach statistical significance (β = 0.11, 95% CI − 0.01 to 0.23, *p* = 0.071). Older age was independently associated with lower HAQ scores, while higher education and socioeconomic status were associated with better HAQ outcomes. Model fit was slightly stronger in the categorical physical activity models (HAQ R² = 0.32; HAPQ R² = 0.18) than in the continuous models (HAQ R² = 0.30; HAPQ R² = 0.14).

**Table 4:** Multivariable linear regression models predicting healthy ageing outcomes

Continuous PA models Outcome = HAQ and HAPQ						
predictor		Model A1: PA continuous (per 100 MET-min/week) β (95% CI)	p-value		HAPQ β (95% CI)	p-value
PA (per 100 MET-min/week)		0.85 (0.35, 1.35)	0.001		0.018 (0.006, 0.030)	0.003
Age		−0.42 (− 0.66, − 0.18)	0.001		−0.004 (− 0.009, 0.001)	0.11
Male(vs. female)		1.2 (− 1.8, 4.2)	0.43		0.05 (− 0.07, 0.17)	0.40
Married (vs. not)		2.0 (− 0.8, 4.8)	0.16		0.06 (− 0.05, 0.17)	0.29
Education: Primary/secondary (vs. none)		2.1 (− 0.9, 5.1)	0.17		0.05 (− 0.07, 0.17)	0.42
Education: above high school (vs. none)		4.1 (0.6, 7.6)	0.021		0.09 (− 0.05, 0.23)	0.20
SES: Middle (vs. low)		3.6 (0.8, 6.4)	0.012		0.07 (− 0.04, 0.18)	0.21
SES: High (vs. low)		6.9 (2.3, 11.5)	0.004		0.12 (0.01, 0.23)	0.033
Model fit: HAQ R² = 0.30; HAPQ R² = 0.14						
Categorical PA models Outcome = HAQ and HAPQ; ref = Low PA						
Predictor	HAQ β (95% CI)			p-value	HAPQ β (95% CI)	p-value
Moderate PA (vs. low)	4.9 (0.8, 9.0)			0.019	0.11 (− 0.01, 0.23)	0.071
High PA (vs. low)	10.8 (5.0, 16.6)			< 0.001	0.21 (0.07, 0.35)	0.004
Age	−0.40 (− 0.64, − 0.16)			0.001	−0.004 (− 0.009, 0.001)	0.10
Male (vs. female)	1.0 (− 2.0, 4.0)			0.51	0.04 (− 0.08, 0.16)	0.52
Married (vs. not)	1.8 (− 1.0, 4.6)			0.21	0.05 (− 0.06, 0.16)	0.37
Education: Primary/secondary (vs. none)	2.0 (− 1.0, 5.0)			0.19	0.05 (− 0.07, 0.17)	0.41
Education: above high school (vs. none)	4.0 (0.5, 7.5)			0.024	0.08 (− 0.06, 0.22)	0.26
SES: Middle (vs. low)	3.4 (0.6, 6.2)			0.017	0.06 (− 0.05, 0.17)	0.28
SES: High (vs. low)	6.6 (2.0, 11.2)			0.005	0.11 (0.00, 0.22)	0.049
≥ 2 chronic conditions (vs. 1)	−3.3 (− 6.4, − 0.2)			0.036	−0.07 (− 0.19, 0.05)	0.26

Model fit: HAQ R² = 0.32; HAPQ R² = 0.18

### Sensitivity and interaction analyses

Results were robust in sensitivity analyses (Table 5). Excluding the top 5% of total physical activity values yielded similar estimates for the association between physical activity and both HAQ and HAPQ. This exclusion was conducted as an additional robustness check and was not part of the standard IPAQ scoring procedure. Use of robust standard errors and additional adjustment for number of chronic conditions and self-rated health did not materially alter the results. Exploratory interaction analyses suggested that the association between physical activity and HAQ may be stronger in women than in men (interaction *p* = 0.04), while evidence for stronger associations in participants aged ≥ 70 years was weaker (interaction *p* = 0.08).

**Table 5:** Sensitivity and exploratory interaction analyses for the association between physical activity and healthy ageing outcomes

A) Sensitivity analyses (continuous PA models)			
Analysis	Outcome	β (per 100 MET-min/wk)	p-value
Primary adjusted model	HAQ total	0.85	0.001
	HAPQ total	0.018	0.003
Excluding extreme PA (top 5%)	HAQ total	0.79	0.002
	HAPQ total	0.017	0.006
Robust SEs (heteroskedasticity-consistent)	HAQ total	0.83	0.002
	HAPQ total	0.018	0.004
Additional adjustment: chronic conditions and self-rated health	HAQ total	0.72	0.004
	HAPQ total	0.015	0.011
B) Subgroup and interaction analyses (exploratory)			
Effect modification test	Outcome	Interpretation	interaction p-value
PA × Sex (women vs. men)	HAQ total	Association appeared stronger in women	0.04
PA × Age group (≥ 70 vs. 60–69)	HAQ total	Association appeared stronger in participants aged ≥ 70 years	0.08

All models were adjusted for age, sex, education, socioeconomic status, marital status, and chronic disease indicators unless otherwise stated

Interaction models included the main effects and the relevant cross-product term

## Discussion

This study examined the association between physical activity and healthy ageing outcomes among older adults with chronic conditions and/or functional vulnerability. Overall, higher levels of self-reported physical activity were associated with better multidimensional healthy ageing scores and more positive ageing perspectives, even after adjustment for key sociodemographic and health-related factors. These findings add to the existing literature by suggesting that physical activity is closely linked with healthy ageing outcomes in a clinically vulnerable older population, although the cross-sectional design does not allow conclusions about causality or direction of effect.

A notable finding was that the majority of participants had low levels of physical activity, alongside moderate overall healthy ageing scores and relatively poorer performance in physical and socio-occupational domains. This pattern is broadly consistent with previous studies showing that older adults with chronic disease, frailty risk, or rehabilitation needs often have lower activity levels and greater functional limitations than healthier populations [14, 15]. Studies from both high-income and middle-income settings have similarly reported a high prevalence of insufficient physical activity among older adults receiving clinical or rehabilitation services [16, 17]. In the present study, however, participants were recruited from a hospital and affiliated outpatient/rehabilitation clinics, which likely represents a population with greater health needs and functional limitations than community-dwelling older adults. This is important when interpreting the findings, because the results may be most applicable to clinically vulnerable older adults rather than to the general older population.

The observed association between total physical activity and the Healthy Ageing Index was strongest for physical functioning/activity participation and socio-occupational functioning. This is in keeping with earlier studies reporting that more physically active older adults tend to have better mobility, independence, and participation in daily and social roles [18, 19]. Longitudinal and cross-sectional studies have also shown that physical activity is associated with better muscle strength, balance, and lower disability burden in later life [20, 21]. At the same time, the present findings should not be interpreted as showing that physical activity directly improved these outcomes. The reverse explanation is also plausible: older adults with better baseline functional status, fewer mobility limitations, and better general health may be more able to engage in physical activity. This interpretation is also compatible with the WHO healthy ageing framework, which emphasizes the dynamic interaction between intrinsic capacity, functional ability, and environmental context rather than a one-way pathway from behaviour to outcome [22].

The weaker associations observed between physical activity and cognitive/psychological wellbeing, compared with the physical and socio-occupational domains, are also noteworthy. Similar domain-specific patterns have been reported in previous research, where associations with physical functioning were stronger than those with psychological outcomes, especially in cross-sectional studies [23, 24]. This may suggest that psychological aspects of healthy ageing are shaped by a broader set of factors, including social support, resilience, multimorbidity, and life-course experiences, rather than by physical activity alone. It may also reflect measurement issues, as both physical activity and healthy ageing were assessed using self-report instruments, which may be more sensitive to perceived functioning than to more subtle cognitive or emotional states.

Physical activity was also positively associated with healthier ageing perspectives, particularly in the domains of lifestyle change and social relationship change. These findings are broadly consistent with previous studies suggesting that physically active older adults tend to report more positive beliefs about ageing, greater self-efficacy, and stronger perceived control over adaptation to health-related changes [25, 26]. Research on ageing perceptions has further shown that positive views of ageing are associated with healthier behaviours, better psychological wellbeing, and more favourable functional outcomes over time [27]. However, the direction of this relationship is likely to be bidirectional. Just as physical activity may be associated with more adaptive ageing perspectives, older adults with more positive beliefs about ageing and greater confidence in their ability to cope may be more likely to remain active. Accordingly, the present findings should be interpreted as evidence of a positive relationship between physical activity and ageing perspectives, rather than evidence that one necessarily causes the other.

The graded differences observed across IPAQ physical activity categories, with higher healthy ageing and perspective scores among those in the higher activity groups, are also consistent with previous population-based studies that have reported progressively better health and functional outcomes with increasing physical activity [28]. However, in the present study these findings should be interpreted cautiously. The IPAQ categories were based on cross-sectional self-reported behaviour measured at a single time point and therefore cannot establish a true dose–response relationship. In addition, the high physical activity group represented a relatively small minority of the sample, which reduces the stability of estimates and limits the strength of any inference based on that group. It is therefore more appropriate to describe these findings as showing a graded cross-sectional association rather than definitive dose–response evidence.

In the adjusted analyses, the association between physical activity and both healthy ageing outcomes and ageing perspectives remained after accounting for age, sex, education, socioeconomic status, marital status, and chronic disease indicators. This is consistent with earlier work suggesting that the relationship between physical activity and later-life wellbeing is not explained solely by basic sociodemographic or health-status differences [29, 30]. At the same time, the observed associations of older age, lower education, and lower socioeconomic status with poorer healthy ageing outcomes are also well documented in the literature [31, 32]. Taken together, these findings suggest that physical activity should be understood within a broader context of social and structural determinants of ageing rather than as an isolated behavioural factor.

Several limitations should be considered when interpreting these findings. First, the cross-sectional design precludes causal inference and does not allow temporal ordering of physical activity and healthy ageing outcomes to be determined. Better functional capacity and health status may facilitate physical activity participation just as much as physical activity may be associated with better health. Second, physical activity was assessed using the self-reported International Physical Activity Questionnaire, which is vulnerable to recall error and social desirability bias and may overestimate activity levels relative to objective measures. Third, healthy ageing and ageing perspectives were measured using questionnaire-based instruments. Although the HAQ and HAPQ provide useful multidimensional and person-centred information, they may reflect perceived health, expectations, and cultural interpretations of ageing in addition to objective functional or clinical status. Fourth, the sample was drawn from a hospital and affiliated rehabilitation clinics, which limits external validity. Participants in such settings are likely to have greater disease burden and functional limitations than healthier community-dwelling older adults. Finally, although sensitivity analyses supported the robustness of the main findings, some comparisons, particularly those involving the high physical activity group, should be interpreted cautiously because of the relatively small number of participants in that category.

Despite these limitations, the study has several strengths. It focuses on an under-studied group of older adults with chronic conditions and functional vulnerability, uses multidimensional measures aligned with contemporary healthy ageing frameworks, and examines not only healthy ageing status but also ageing perspectives. These strengths provide a broader view of how physical activity relates to both functional and psychosocial aspects of ageing in a middle-income setting.

Overall, the findings suggest that among older adults with chronic conditions and functional vulnerability, higher self-reported physical activity tends to cluster with better healthy ageing outcomes and more positive ageing perspectives. Clinically, this supports the relevance of promoting feasible and context-appropriate physical activity within healthcare and rehabilitation settings, while recognizing that such strategies may need to be tailored to individuals’ underlying functional capacity, comorbidity burden, and social circumstances. Future longitudinal studies using objective physical activity measures and clearer characterization of functional vulnerability are needed to clarify the direction and mechanisms of these associations and to determine whether changes in physical activity are followed by changes in healthy ageing outcomes over time.

## Conclusion

In conclusion, this study found that higher levels of physical activity were positively associated with both multidimensional healthy ageing and more positive perspectives on healthy ageing among older adults with chronic conditions and/or functional vulnerability. Physical activity was consistently associated with functional ability, socio-occupational participation, and lifestyle and social-related ageing perspectives, even after adjustment for key sociodemographic and health-related factors. Participants in the moderate- and high-activity groups also tended to have better healthy ageing outcomes than those in the low-activity group, although these graded differences should be interpreted cautiously given the cross-sectional design and self-reported measurement of physical activity. Overall, the findings suggest that physical activity is an important correlate of healthy ageing in vulnerable older populations, particularly in middle-income countries experiencing rapid population ageing. These results support the relevance of promoting feasible and context-appropriate physical activity within healthcare, rehabilitation, and community-based settings, while recognizing that causal inferences cannot be drawn from the present study. Future longitudinal and interventional research using objective physical activity measures and clearer characterization of functional vulnerability is needed to clarify the direction, mechanisms, and practical implications of these associations.

## Data Availability

The datasets used and/or analyzed during the current study available from the corresponding author on reasonable request. The entire dataset is in Farsi language. The Data can be available in English language for the readers and make available from the corresponding author on reasonable request.
